# Species-Specific Outcome in the Competition for Nitrogen Between Invasive and Native Tree Seedlings

**DOI:** 10.3389/fpls.2019.00337

**Published:** 2019-03-29

**Authors:** Andrea Bueno, Karin Pritsch, Judy Simon

**Affiliations:** ^1^Plant Interactions Ecophysiology Group, Department of Biology, University of Konstanz, Konstanz, Germany; ^2^Institute of Biochemical Plant Pathology, Helmholtz Zentrum München, Deutsches Forschungszentrum für Gesundheit und Umwelt GmbH, Neuherberg, Germany

**Keywords:** inorganic nitrogen, nitrogen pools, nitrogen uptake, organic nitrogen, plant competition, woody invaders

## Abstract

The outcome of competition for nitrogen (N) between native and invasive tree species is a major concern when considering increasing anthropogenic N deposition. Our study investigated whether three native (i.e., *Fagus sylvatica, Quercus robur*, and *Pinus sylvestris*) and two invasive woody species (i.e., *Prunus serotina* and *Robinia pseudoacacia*) showed different responses regarding morphological and physiological parameters (i.e., biomass and growth indices, inorganic vs. organic N acquisition strategies, and N allocation to N pools) depending on the identity of the competing species, and whether these responses were mediated by soil N availability. In a greenhouse experiment, tree seedlings were planted either single or in native-invasive competition at low and high soil N availability. We measured inorganic and organic N acquisition using ^15^N labeling, total biomass, growth indices, as well as total soluble amino acid-N and protein-N levels in the leaves and fine roots of the seedlings. Our results indicate that invasive species have a competitive advantage via high growth rates, whereas native species could avoid competition with invasives via their higher organic N acquisition suggesting a better access to organic soil N sources. Moreover, native species responded to competition with distinct species- and parameter-specific strategies that were partly mediated by soil N availability. Native tree seedlings in general showed a stronger response to invasive *P. serotina* than *R. pseudoacacia*, and their strategies to cope with competition reflect the different species’ life history strategies and physiological traits. Considering the responses of native and invasive species, our results suggest that specifically *Q. robur* seedlings have a competitive advantage over those of *R. pseudoacacia* but not *P. serotina*. Furthermore, native and invasive species show stronger responses to higher soil N availability under competition compared to when growing single. In conclusion, our study provides insights into the potential for niche differentiation between native and invasive species by using different N forms available in the soil, the combined effects of increased soil N availability and competition on tree seedling N nutrition, as well as the species-specific nature of competition between native and invasive tree seedlings which could be relevant for forest management strategies.

## Introduction

Biological plant invasions have consequences on plant interactions in native communities, thus severely affecting ecosystems in the future given that plant community composition and structure are largely determined by the outcome of plant–plant interactions such as facilitation and/or competition for limiting resources (Goldberg and Barton, 1992). Considering that nutrients, especially nitrogen (N), limit woody plant growth (Körner, 2003; Miller et al., 2007; Millard and Grelet, 2010) and that resource acquisition and internal allocation play an important role in achieving maximum growth and reproductive fitness in plants, particularly long-living woody species, competition for N between native and non-native woody species is of great relevance. Although evidence points in the direction that species in native plant communities have evolved different strategies to avoid competition for N via preference of different N sources to effectively avoid competition via niche differentiation (Näsholm et al., 2009; Hodge and Fitter, 2013; Simon et al., 2017), via N acquisition at different times during the growing season (Simon et al., 2017), and/or via the interaction with soil microorganisms to enhance N acquisition (e.g., Hodge and Fitter, 2013), these strategies might no longer be effective in competition with non-native plant species because of their higher competitive ability compared to native species (Gioria and Osborne, 2014). Considering their higher capacity to exploit limited resources, better resource use efficiency, inhibition of growth, as well as establishment of potential competitors (D´Antonio and Vitousek, 1992; Gioria and Osborne, 2014), non-native plant species have a large potential to become invasive (Keller et al., 2011) and as a result induce profound changes in forest ecosystem structure and functioning (Holmes et al., 2009; Vilà et al., 2011; Aerts et al., 2017).

The outcome of plant interactions is not only determined by biotic interactions but also shifts depending on the environmental conditions (Aschehough et al., 2016). Abiotic factors, such as light availability (Simon et al., 2014), water availability (Fotelli et al., 2001, 2002), air temperature (Fotelli et al., 2005) as well as soil N availability (e.g., Li et al., 2015) influence the competition for N between plants, thereby shifting the outcome of plant–plant competition. For example, nitrate acquisition of sycamore maple (*Acer pseudoplatanus* L.) decreased when competing with European beech (*Fagus sylvatica* L.) compared to intraspecific competition at high but not low soil N availability suggesting that the response to competition for N is mediated by soil N availability (Li et al., 2015). Furthermore, plant–plant competition and soil N availability showed combined effects on plant N nutrition by inducing changes in N pools in the fine roots such as total soluble protein-N and total amino acid-N (Li et al., 2015). Overall, the interplay between interspecific competition for N and varying environmental conditions can impact on plant N acquisition and N nutrition with consequences for plant growth and survival, which in turn may affect plant community composition and structure (Aerts et al., 2017). However, in general, non-woody species are investigated preferably in plant interaction studies, thus the understanding of the mechanisms underlying tree interactions is rather scarce (Trinder et al., 2013; Pommerening and Sánchez Meador, 2018), although resource acquisition and internal allocation are particularly important for resource-storing and -remobilizing in long-living trees.

Woody invasive species are a potential threat for forest habitats throughout Europe (Campagnaro et al., 2018), and studies indicate their highly competitive characteristics. For example, root competition with seedlings of *Prunus serotina* and *Robinia pseudoacacia* – the most important invaders in natural and semi-natural woodlands in Central Europe (Halarewicz et al., 2017; Campagnaro et al., 2018) – decreased total biomass and modified biomass allocation of seedlings of the native species *Quercus robur* and *Carpinus betulus* (i.e., by increased root biomass at the expense of aboveground production) (Kawaletz et al., 2013, 2014). Furthermore, competition intensity increased more over time for the native species when competing with the two invasive species compared to intraspecific competition or competition among natives (Kawaletz et al., 2013, 2014). On the other hand, competition with *P. serotina* stimulated the height growth of seedlings of *Q. petraea*, suggesting a potential for species-specific competitive effects (Robakowski and Bielinis, 2011).

Although these studies stress the competitive effects of invasive *P. serotina* and *R. pseudoacacia* on the growth of native species, the understanding on the interactions between native and invasive woody species with regard to N cycling in forest ecosystems depending on abiotic stressors (i.e., soil N availability) is very limited. Soil N availability is expected to rise in terrestrial ecosystems of Central Europe as a result of increased atmospheric deposition (Rennenberg et al., 2009; Waldner et al., 2014), which will also impact on the outcome of invasion processes in plant communities (Bradley et al., 2010; Littschwager et al., 2010; Luo et al., 2014). Therefore, it is key to understand the processes of interspecific competition for N between native and invasive tree species, as well as the effects of soil N availability on the outcome of this competition with regard to plant N acquisition and N nutrition to predict the future dynamics of forest ecosystems. Thus, we conducted a greenhouse experiment using different native and invasive tree species to investigate whether native and invasive tree species show species-specific responses in terms of growth, N acquisition strategies and N allocation to N pools depending on the identity of their competitor, and whether those responses are influenced by increased soil N availability. The native species (i.e., *F. sylvatica* L., *Q. robur* L., and *Pinus sylvestris* L.), represent the most abundant species in Central European forests (Ellenberg and Leuschner, 2010). The two invasive species (i.e., *P. serotina* Ehrh. and *R. pseudoacacia* L.) are among the top five invasive tree species in Central European forests (Campagnaro et al., 2018). The selected species differ in their physiology and growth strategies: *F. sylvatica* and *Q. robur* are late successional, broadleaved species differing in their drought tolerance – i.e., *Q. robur* is drought tolerant, found on fertile, loamy-clay soils, whereas *F. sylvatica* is sensitive to drought, and is mostly found on moderately fertile, calcareous soils (Cavin et al., 2013; Eaton et al., 2016; Houston et al., 2016a). *P. sylvestris* is an evergreen conifer with needles present throughout the year functioning as N storage organs (Millard and Grelet, 2010), as opposed to the broadleaved species which lose their leaves in the autumn season. Regarding the invasive species, *P. serotina* produces cyanogenic compounds that negatively affect other plant species (Csiszár, 2009) by reducing their germination and growth rates (Robakowski et al., 2016), while *R. pseudoacacia* can fix atmospheric N_2_ via symbiotic bacteria in root nodules and is potentially dominant at nutrient poor sites (Danso et al., 1995; Vítková et al., 2017). Moreover, we have chosen seedlings of uniform age to incorporate the aspect of the different demands for nitrogen between species according to their growth strategies (Reich, 2014). These differences in physiological aspects between the species may result in different response strategies to interspecific competition and also shift with increasing soil N availability. We hypothesized that: (1) Invasive species have traits that allow them to successfully grow and establish compared to native species. (2) Higher soil N availability has a positive effect on the growth and N strategies of invasive species compared to native species. (3) Native tree species respond species-specific depending on the identity of the invasive competitor species. (4) Invasive species respond differently to different native competitor species. (5) Seedling responses to competition are mediated by soil N availability.

## Materials and Methods

### Plant Material and Growth Conditions

We chose three tree species native to and widely found in Central European forests: (i) European beech (*F. sylvatica* L., Fagaceae), (ii) pedunculate oak (*Q. robur* L., Fagaceae), and (iii) Scots pine (*P. sylvestris* L., Pinaceae). As invasive species we chose two tree species native to North America that were introduced to Europe in the 17*^th^* century as ornamental plants and later widely planted for restoration and reforestation purposes: (i) black cherry (*P. serotina* Ehrh., Rosaceae), and (ii) black locust (*R. pseudoacacia* L., Leguminosae) (Starfinger et al., 2003; Vítková et al., 2017). From here on, species used in this study will be referred to by their genus, i.e., *Fagus, Quercus, Pinus, Prunus* and *Robinia*. For all species, 1-year-old seedlings were purchased from a commercial tree nursery (Müller Münchehof Pflanzen GmbH, Seesen/Münchehof, Germany) and planted in different competition regimes (one or two seedlings per pot, see section “Experimental Design”) in a 1:1 mixture of sand and vermiculite in 3 L plastic pots (25 cm × 12 cm) at the end of November 2015. Pots overwintered outdoors and were brought into the greenhouse in early March 2016. For the next 10 days, pots were watered regularly and sufficiently with tap water. From mid-March, pots received 100 ml of a low N nutrient solution (see solution composition below) every second day as watering until the end of leaf development (early May) when the soil N availability treatments started (see section “Experimental Design”). The pots were exposed to natural light conditions and day/night regime. The average air temperature was 19.3 ± 4.0°C/16.0 ± 3.8°C (day/night, mean ± standard deviation). The average relative humidity was 54.7 ± 13.0%/63.4 ± 10.1% (day/night, mean ± standard deviation).

### Experimental Design

The experiment was conducted in a fully orthogonal design with two factors, “soil N availability” (i.e., low or high) and “competitor identity” (i.e., native and invasive species in interspecific competition). Seedlings were planted in interspecific competition between native and invasive species (i.e., one seedling of a native species and one seedling of an invasive species per pot). Interspecific competition pots were established for every combination of native and invasive species. Furthermore, seedlings were planted without competition (i.e., one seedling per pot) to compare parameters between species and to determine the species general strategies and their responses to soil N availability without competition. For each species, a total of 24 pots were established as single seedlings as well as for each combination of competitor identity, summing up to a total of 264 pots. In early May, pots were assigned to either the low or high soil N availability treatment (i.e., *n* = 12 per combination of species, competitor identity, and soil N availability treatment). Pots were irrigated every second day with 100 ml of either low N or high N artificial nutrient solution mimicking a low (Dannenmann et al., 2009) or high soil N field site (Stoelken et al., 2010) for 6 weeks. Tests prior to the experiment showed that 100 ml of water were sufficient under the moderate air temperatures that prevailed early in the experiment. When temperatures increased in late May, additional irrigation was provided (see below). The artificial low N nutrient solution consisted of 100 μM KNO_3_, 90 μM CaCl_2_^∗^2H_2_O, 70 μM MgCl_2_^∗^6H_2_O, 50 μM KCl, 24 μM MnCl_2_^∗^4H_2_O, 20 μM NaCl, 10 μM AlCl_3_, 7 μM FeSO_4_^∗^7H_2_O, 6 μM K_2_HPO_4_, 1 μM NH_4_Cl, 25 μM glutamine, and 25 μM arginine. The artificial high N nutrient solution consisted of 20 μM Al_2_(SO_4_)_3_, 75 μM CaCl_2_^∗^2H_2_O, 4 μM FeCl_3_^∗^6H_2_O, 14 μM KCl, 10 μM MnCl_2_^∗^4H_2_O, 40 μM MgCl_2_^∗^6H_2_O, 4.5 μM Na_2_HPO_4_, 20 μM NaCl, 50 μM NH_4_Cl, 300 μM KNO_3_, 100 μM glutamine, and 100 μM arginine. Additionally, from the end of May, pots were irrigated with tap water every second day (i.e., alternating with the days when the low/high soil N solutions were applied) to avoid drought stress related to increased air temperatures and solar radiation until the ^15^N uptake experiments and final harvest commenced in mid-June.

### ^15^N Uptake Experiments

To quantify net inorganic (i.e., ammonium and nitrate) and organic (i.e., glutamine and arginine) N uptake capacity of the fine roots of the seedlings, the ^15^N enrichment technique was used as described by Gessler et al. (1998) and modified by Simon et al. (2010) prior to the harvest of the seedlings. Seedlings were carefully removed from the pots, and their roots thoroughly washed with tap water to remove adherent substrate particles. Fine roots still attached to the seedlings were then incubated for 2 h in the artificial soil solution (*n* = 4–6) according to their treatment (either low or high N as described above) containing all four N sources, but with only one of them labeled as either ^15^${NH}_{4}^{+}$, ^15^${NO}_{3}^{-}$, ^13^C/^15^N-glutamine, or ^13^C/^15^N-arginine. The remaining roots were carefully wrapped in wet tissue to avoid desiccation. After incubation, the fine roots were cut off and washed twice in 0.5 M CaCl_2_ to remove the incubation solution from the root surface. The fresh weight was determined, followed by oven-drying at 60°C for 48 h and determination of the dry weight. Amino acids were ^13^C/^15^N-labeled to determine whether they are taken up as intact molecules (Simon et al., 2011). Controls with no ^15^N or ^13^C label were included to account for natural abundance of ^15^N and ^13^C in the fine roots. Incubation took place between 10 am and 2 pm to avoid diurnal variation in net N uptake capacity (Gessler et al., 2002).

### Harvest and Quantification of Plant Growth Indices

To calculate relative growth rates (RGRs), 3 to 4 pots per combination of species and competitor identity were harvested after leaf development and before commencing the soil N availability treatments to determine initial seedling biomass. Initial total seedling biomass (mean ± SD) was for *Fagus* 1.65 ± 0.53 g dw, for *Quercus* 6.43 ± 4.50 g dw, for *Pinus* 3.05 ± 1.16 g dw, for *Prunus* 10.62 ± 7.91 g dw, and for *Robinia* 4.23 ± 3.00 g dw (mean ± SD). Initial stem length was 21.7 ± 3.8 cm for *Fagus*, 33.4 ± 5.2 cm for *Quercus*, 29.2 ± 5.3 cm for *Pinus*, 63.8 ± 16.3 cm for *Prunus*, and 54.8 ± 8.4 cm for *Robinia*. Subsequent to the ^15^N uptake experiments, all remaining seedlings were separated into leaves, stems, and roots. After determining their fresh weight, all organs were oven-dried at 60°C for 48 h, and their dry weight was determined. On the final harvest, a subset of 8 to 10 representative leaves was collected from each seedling and leaf area was measured (LI-3100C Area Meter, LI-COR, Lincoln, NE, United States) as well as fresh and dry weight determined to calculate specific leaf area (SLA). Based on Liu and van Kleunen (2017), a subset of fine roots was collected, stained, scanned, and their total length measured (WinRhizo 2012, Regent Instruments, Inc., Quebec City, QC, Canada) to calculate specific root length (SRL) before oven-drying and subsequent dry weight determination. Additionally, leaf and fine root samples were collected from each seedling for quantification of total soluble amino acid and total soluble protein levels, shock-frozen in liquid N_2_ immediately after determining their fresh weight, and stored at -80°C until further analyses. Root:shoot ratio was calculated as the ratio between total root biomass and the combined biomass of stem and leaves. RGR was calculated for each seedling following the formula: RGR = (ln b_2_ – ln b_1_) ^∗^ t^-1^, where b_1_ is total seedling biomass in grams at initial harvest, b_2_ is total seedling biomass in grams at the final harvest, and t is the time period in days between the initial harvest and the final harvest (Grubb et al., 1996).

### Quantification of Total N and C, ^15^N, and ^13^C in Fine Roots

To quantify total N and C as well as ^15^N and ^13^C enrichment, dried fine root samples were ground to a fine homogenous powder using a ball mill (TissueLyser, Retsch, Haan, Germany). Aliquots of 1.2 to 2.4 mg were weighed into 4 mm × 6 mm tin capsules (IVA Analysentechnik, Meerbusch, Germany) and analyzed with an isotope ratio mass spectrometer (Delta V Advantage, Thermo Electron, Dreieich, Germany) coupled to an elemental analyzer (Euro EA, Eurovector, Milan, Italy). Δ Values were calculated using a laboratory standard (acetanilide) that was part of every sequence in intervals also used in different weights to determine isotope linearity of the system. The laboratory standard was calibrated against several suitable international isotope standards (IAEA, Vienna). Final correction of isotope values was done with several international isotope standards and other suitable laboratory standards which cover the range of ^15^N and ^13^C results. Net N uptake capacity (nmol N g^-1^ fw h^-1^) was calculated based on the incorporation of ^15^N into the fine root according to Gessler et al. (1998): N uptake capacity = ((^15^N*_l_*-^15^N*_c_*)∗N_tot_∗dw∗10^5^)/(MW∗fw∗t)^-1^, where ^15^N*_l_* and ^15^N*_c_* are the atom% of ^15^N in labeled (N*_l_*) and control plants (N*_c_*, natural abundance), respectively, N_tot_ is the total N percentage, MW is the molecular weight (^15^N g mol^-1^), and t represents the incubation time. Net uptake capacity of the amino acids glutamine and arginine was lower based on ^13^C incorporation than on ^15^N incorporation indicating either (1) the degradation of amino acids in the solution or on the root surface, and/or (2) the respiration of amino acid-derived C inside the roots (Simon et al., 2011).

### Quantification of Total Soluble Protein and Total Soluble Amino Acid Levels in Leaves and Fine Roots of Seedlings

Total soluble protein levels in the leaves and fine roots were extracted based on Dannenmann et al. (2009). Aliquots of ∼50 mg ground frozen organ were incubated in 1.5 ml extraction buffer (50 mM Tris-HCl pH 8.0, 1 mM EDTA, 15% (v/v) glycerol, 0.6 mM dithiothreitol, 1% Triton X-100, 2 EDTA-free protease inhibitor cocktail tablets per 100 ml buffer solution) at 4°C for 30 min and subsequently centrifuged for 10 min at 14,000 rpm and 4°C. The extraction was repeated once to increase the yield. Then, 500 μL of the combined supernatant were incubated with 1 ml 10% (v/v) trichloroacetic acid for 10 min at room temperature followed by centrifugation for 10 min at 14,000 rpm and 4°C. The protein pellet was dissolved in 1 ml 1 M KOH. To quantify total soluble protein levels according to Simon et al. (2010), 1 ml of Bradford reagent was added to 50 μL of extract. After 10 min incubating in the dark at room temperature, the absorbance at 595 nm was measured in a spectrophotometer (Ultrospec 3100pro, Amersham Biosciences). Bovine serum albumin (BSA) was used as standard.

To quantify total soluble amino acid levels in the leaves and fine roots, aliquots of ∼50 mg of frozen organ were extracted in 200 μL Hepes-buffer (5 mM EGTA, 20 mM HEPES, 10 mM NaF) and 1 ml 3.5:1.5 (v:v) methanol/chloroform, according to Winter et al. (1992). After 30 min incubation on ice, 600 μL of distilled water were added and the samples centrifuged for 5 min at 14,000 rpm and 4°C. The addition of distilled water was repeated once to increase the yield. For the quantification, according to Liu et al. (2005), 50 μL ninhydrin solution was added to a 50 μL aliquot of the combined extract and boiled for 30 min. The ninhydrin solution consisted of a 1:1 mixture of solution A (i.e., 3.84 g citric acid, 0.134 g SnCl_2_, and 40 ml 1 M NaOH, filled up to 100 ml with distilled water at pH 5), and solution B (i.e., 4 g ninhydrin in 100 ml ethylene-glycol-monomethyl-ether). After cooling to room temperature, 1 ml 50% isopropanol was added to the extract and incubated for 15 min. The absorption was measured at 570 nm in a spectrophotometer (Ultrospec 3100pro, Amersham Biosciences). L-Glutamine was used as standard.

### Statistical Analyses

For all species, differences between treatment levels (i.e., competitor identity and low/high soil N availability) were tested (alpha level of 0.050) for total biomass, root:shoot ratio, SLA, SRL, RGR, total soluble amino acid, and total soluble protein contents in the leaves and fine roots, as well as inorganic and organic net N uptake capacity by performing Permutational ANOVAs (PERMANOVA) based on a Euclidean resemblance matrix between samples (Anderson et al., 2008). We performed two-way PERMANOVAs using “soil N availability” and “competitor identity” as orthogonal factors. The factor “soil N availability” had two levels: (i) low N and (ii) high N. The factor “competitor identity” had two levels for native species: (i) competition with *Prunus* and (ii) competition with *Robinia*; and three levels for the invasive species: (i) competition with *Fagus*, (ii) competition with *Quercus*, and (iii) competition with *Pinus*. When a significant interaction between factors was found, *post hoc* PERMANOVA pair-wise comparisons were performed. To test for differences between species in terms of SLA, SRL, RGR, total soluble amino acid and total soluble protein contents in the leaves and roots, as well as net uptake capacity of the four N sources, two-way PERMANOVAs were performed on the single seedlings data (i.e., no competition), using “species” and “soil N availability” as factors. To test for significant differences between low and high soil N availability for each species growing in absence of competition, Mann–Whitney *U*-tests were performed for all measured parameters. Furthermore, to test for preferences in net N uptake capacity, one-way PERMANOVAs were performed for each combination of species and competitor identity using “N source” as factor at both levels of soil N availability. All PERMANOVA analyses were performed using PRIMER 6.0 with the PERMANOVA+ add-on (PRIMER-E, Ltd., Plymouth, United Kingdom), while Mann–Whitney-*U* tests were performed using SigmaPlot 14.0 (Systat Software, San Jose, CA, United States).

## Results

### Comparison Between Species and Responses to Soil N Availability Without Competition

#### General Differences in N Acquisition and Growth Strategies Between Tree Species

Seedlings of invasive species had a significantly higher total biomass and RGR than native seedlings, with *Prunus* having the largest total biomass among all species considered (Table 1 and Supplementary Table 1). Moreover, invasive *Robinia* had significantly higher SLA and SRL than both *Prunus* and the three native species (*Fagus*, *Quercus*, and *Pinus*) (Table 1 and Supplementary Table 1). Similarly, *Robinia* had in general significantly higher levels of total soluble amino acid-N and protein-N in the leaves and fine roots than all other investigated species (Table 1 and Supplementary Table 2). Regarding N acquisition, inorganic N net uptake capacity did not differ significantly between species. However, seedlings of native tree species had significantly higher organic N net uptake capacity than seedlings of invasive tree species (Table 1 and Supplementary Table 3).

**Table 1:** Differences in total biomass, growth indices, inorganic and organic N net uptake capacity, and N metabolite levels between single grown seedlings of *Fagus sylvatica*, *Quercus robur*, *Pinus sylvestris*, *Prunus serotina*, and *Robinia pseudoacacia*.

	Biomass and growth indices				N net uptake capacity				N metabolites			
		
									Total soluble amino		Total soluble	
			
									acid-N		protein-N	
			
Total	Root:shoot	RGR	SLA	SRL	${NH}_{4}^{+}$	${NO}_{3}^{-}$	Gln-N	Arg-N	Leaves	Fine	Leaves	Fine
biomass	ratio									roots		roots
*Prunus* >	*Pinus* >	*Prunus*,	*Robinia* >	*Robinia* >	n.s.	n.s.	*Fagus*,	*Fagus*,	*Robinia* >	*Prunus*,	*Robinia*,	*Robinia* >
*Robinia* >	*Quercus* >	*Robinia* >	*Fagus* >	*Fagus*,			*Pinus* >	*Quercus*,	*Prunus* >	*Robinia* >	*Fagus*,	*Fagus*,
*Quercus* >	*Prunus* >	*Fagus*,	*Quercus* >	*Quercus*,			*Prunus*,	*Pinus* >	*Fagus*,	*Quercus*,	*Pinus* >	*Quercus*,
*Fagus*,	*Fagus* >	*Quercus*,	*Prunus*	*Pinus*,			*Robinia*	*Prunus*,	*Quercus*,	*Pinus*	*Prunus*	*Pinus*,
*Pinus*	*Robinia*	*Pinus*		*Prunus*				*Robinia*	*Pinus*			*Prunus*


*Species A > species B, seedlings of species A had significantly higher values than seedlings of species B; n.s., no significant differences between species. Total biomass (g), root:shoot ratio, ratio of belowground biomass to aboveground biomass; RGR, relative growth rate (g dw g^-*1*^ dw d^-*1*^); SLA, specific leaf area (cm^*2*^ g^-*1*^ dw); SRL, specific root length (cm g^-*1*^ dw); N net uptake capacity (nmol N g^-*1*^ fw h^-*1*^); ${NH}_{4}^{+}$, ammonium; ${NO}_{3}^{-}$, nitrate; Gln-N, glutamine; Arg-N, arginine; total soluble amino acid-N (mg amino acid-N g^-*1*^ dw); total soluble protein-N (mg protein-N g^-*1*^ dw).*

#### Individual Species Responses to High Compared to Low Soil N Availability

Increased soil N availability had neither a significant effect on the total biomass nor the growth indices of the single grown species, except for a significantly lower root:shoot ratio of *Robinia* (Table 2A and Supplementary Table 4). However, inorganic and organic N net uptake capacity increased significantly for all species with high compared to low soil N availability following species-specific patterns. More specifically, an increase in net uptake capacity was shown for the native species for ammonium, nitrate, and arginine-N of *Fagus*, for ammonium and arginine-N of *Quercus*, and for all N forms of *Pinus*. Both invasive species had an increased ammonium and arginine-N net uptake capacity with higher soil N availability (Table 2A and Supplementary Table 5). Furthermore, the changes in N metabolites levels in the leaves and fine roots at high compared to low soil N availability were also species-specific: *Fagus* had higher total soluble protein-N content in the leaves, *Quercus* had lower total soluble amino acid-N content in the leaves, and *Prunus* had lower total soluble amino acid-N content in the leaves and fine roots, while for *Pinus* and *Robinia* the N metabolite content in the leaves and fine roots did not differ significantly between low and high soil N availability (Table 2A and Supplementary Table 6). No other significant differences were found between soil N availability treatments in the single grown seedlings.

**Table 2:** Effects of increased soil N availability on total biomass, growth indices, inorganic and organic N net uptake capacity, and N metabolite levels on **(A)** seedlings growing single and **(B)** seedlings growing in competition of *Fagus sylvatica, Quercus robur, Pinus sylvestris, Prunus serotina*, and *Robinia pseudoacacia*.

	Biomass and growth indices					N net uptake capacity				N metabolites			
				
										Total soluble		Total soluble	
										amino acid-N		protein-N	
				
	Total	Root:shoot	RGR	SLA	SRL	${NH}_{4}^{+}$	${NO}_{3}^{-}$	Gln-N	Arg-N	Leaves	Fine	Leaves	Fine
	biomass	ratio									roots		roots
**(A) Seedlings growing single**													
*Fagus*	n.s.	n.s.	n.s.	n.s.	n.s.	↑	↑	n.s.	↑	n.s.	n.s.	n.s.	↑
*Quercus*	n.s.	n.s.	n.s.	n.s.	n.s.	↑	n.s.	n.s.	↑	↓	n.s.	n.s.	n.s.
*Pinus*	n.s.	n.s.	n.s.	n.s.	n.s.	↑	↑	↑	↑	n.s.	n.s.	n.s.	n.s.
*Prunus*	n.s.	n.s.	n.s.	n.s.	n.s.	↑	n.s.	n.s.	↑	n.s.	n.s.	n.s.	n.s.
*Robinia*	n.s.	↓	n.s.	n.s.	n.s.	↑	n.s.	n.s.	↑	n.s.	n.s.	n.s.	n.s.

**(B) Seedlings growing in competition**													
*Fagus*	n.s.	n.s.	n.s.	n.s.	↓	↑	n.s.	n.s.	↑	↓	↓	n.s.	n.s.
*Quercus*	n.s.	n.s.	↓	n.s.	n.s.	↑	n.s.	↑	↑	n.s.	↓(b)	n.s.	↓
*Pinus*	n.s.	n.s.	↓	n.s.	n.s.	↑	n.s.	n.s.	↑	↓	n.s.	n.s.	n.s.
*Prunus*	n.s.	n.s.	n.s.	n.s.	↓	↑	n.s.	↑	↑	↓	↓	↓	n.s.
*Robinia*	n.s.	n.s.	n.s.	n.s.	n.s.	↑	↑	↑(a)	↑	↓	↓(c)	n.s.	↓


*↑, significant increase with increased soil N availability; ↓, significant decrease with increased soil N availability; n.s., no significant differences between high and low soil N availability. Total biomass (g), root:shoot ratio, ratio of belowground biomass to aboveground biomass; RGR, relative growth rate (g dw g^-*1*^ dw d^-*1*^); SLA, specific leaf area (cm^*2*^ g^-*1*^ dw); SRL, specific root length (cm g^-*1*^ dw); N net uptake capacity (nmol N g^-*1*^ fw h^-*1*^); ${NH}_{4}^{+}$, ammonium; ${NO}_{3}^{-}$, nitrate; Gln-N, glutamine; Arg-N, arginine; total soluble amino acid-N (mg amino acid-N g^-*1*^ dw); total soluble protein-N (mg protein-N g^-*1*^ dw). (a) Only in competition with *Fagus* and *Pinus.* (b) Only in competition with *Prunus.* (c) Only in competition with *Fagus* and *Quercus.* No letter: effect of soil N availability regardless of competitor identity.*

### Effects of Competition on Native and Invasive Tree Species

#### Native Tree Species – Differences in the Response to Competitor Identity Regarding Total Biomass, Growth Indices, N Acquisition, and N Pools in the Leaves and Fine Roots

**FIGURE 1:**
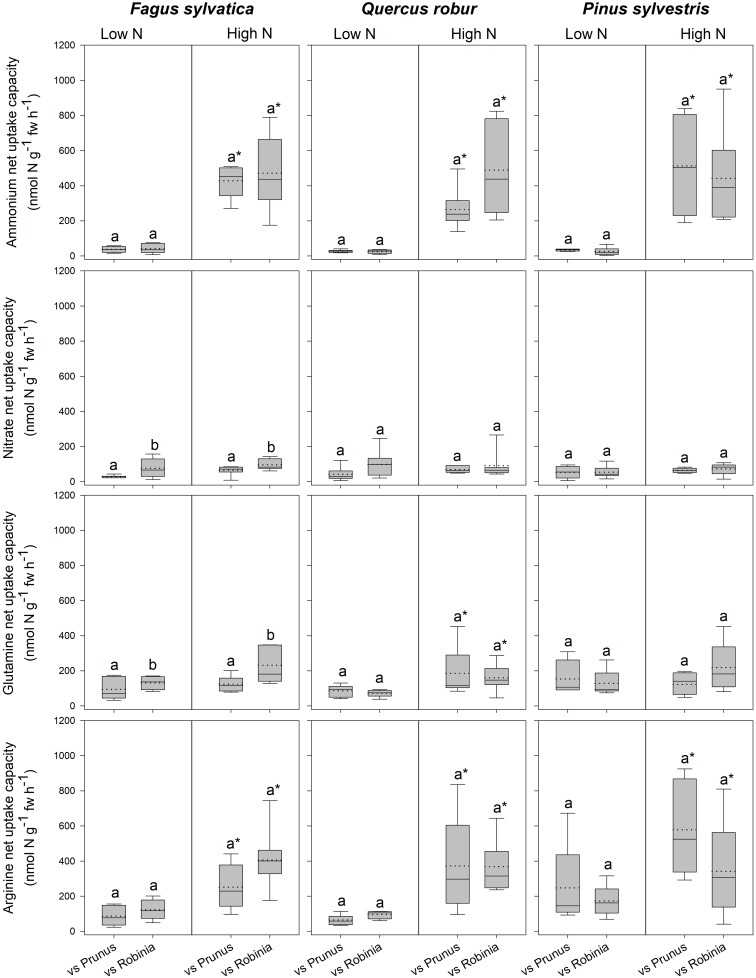
Ammonium, nitrate, glutamine-N, and arginine-N net uptake capacity (nmol N g^-1^ fw h^-1^) by fine roots of *Fagus sylvatica*, *Quercus robur*, and *Pinus sylvestris* seedlings at low and high soil N availability under different competition regimes. vs. Prunu*s* = competition with *Prunus serotina*; vs. Robinia = competition with *Robinia pseudoacacia*. Box plots show mean (dotted line) and median (continuous line). Different letters indicate significant differences between competition regimes within a specific soil N availability treatment, and asterisks indicate significant differences between soil N availability treatments detected using permutational analysis of variance (*p* < 0.05).

*Fagus* seedlings grown in competition with *Prunus* had significantly lower nitrate and glutamine-N net uptake capacity as well as RGR, regardless of soil N availability than when grown with *Robinia*, while no other parameter differed significantly with different competitor species (Figure 1, Table 3 and Supplementary Tables 7, 8A). Unlike *Fagus*, for *Quercus* seedlings inorganic and organic N net uptake capacity did not differ between different competitor identities (Figure 1 and Table 3). However, seedlings of *Quercus* had significantly lower total biomass under competition with *Prunus* compared to under competition with *Robinia*, regardless of soil N availability (Table 3 and Supplementary Table 7). Moreover, total soluble amino acid-N content in the fine roots of *Quercus* seedlings was also significantly lower when grown in competition with *Prunus* compared to *Robinia* only at high soil N availability (Table 3 and Supplementary Tables 8A,B). For *Quercus* seedlings no differences were found between different competitor identities regarding all other parameters (Figure 1, Table 3 and Supplementary Tables 7, 8A). For *Pinus* seedlings the only difference between competitor identities was a significantly higher SRL for seedlings competing with *Prunus* compared to those competing with *Robinia* (Figure 1, Table 3 and Supplementary Tables 7, 8A).

**Table 3:** Effects of competitor identity on total biomass, growth indices, inorganic and organic N net uptake capacity, and N metabolite levels of *Fagus sylvatica*, *Quercus robur*, *Pinus sylvestris*, *Prunus serotina*, and *Robinia pseudoacacia* seedlings.

Biomass and growth índices						N net uptake capacity				N metabolites			
				
										Total soluble		Total soluble	
										amino acid-N		protein-N	
				
	Total biomass	Root: shoot ratio	RGR	SLA	SRL	${NH}_{4}^{+}$	${NO}_{3}^{-}$	Gln-N	Arg-N	Leaves	Fine roots	Leaves	Fine roots
*Fagus*	n.s.	n.s.	*Prunus* <*Robinia*	n.s.	n.s.	n.s.	*Prunus* <*Robinia*	*Prunus* <*Robinia*	n.s.	n.s.	n.s.	n.s.	n.s.
*Quercus*	*Prunus* <*Robinia*	n.s.	n.s.	n.s.	n.s.	n.s.	n.s.	n.s.	n.s.	n.s.	*Prunus* <*Robinia* (a)	n.s.	n.s.
*Pinus*	n.s.	n.s.	n.s.	n.s.	*Robinia* <*Prunus*	n.s.	n.s.	n.s.	n.s.	n.s.	n.s.	n.s.	n.s.
*Prunus*	n.s.	n.s.	n.s.	*Fagus* <*Quercus*	n.s.	n.s.	n.s.	n.s.	n.s.	n.s.	n.s.	n.s.	n.s.
*Robinia*	n.s.	n.s.	n.s.	*Fagus* <*Quercus*, *Pinus*	n.s.	n.s.	n.s.	*Quercus* <*Fagus*, *Pinus* (a)	n.s.	n.s.	*Quercus*, *Pinus* <*Fagus* (b)	n.s.	n.s.


*Species A < species B, seedlings competing with species A had significantly lower values than seedlings competing with species B; n.s., no significant differences between competitor identities. Total biomass (g), root:shoot ratio, ratio of belowground biomass to aboveground biomass; RGR, relative growth rate (g dw g^-*1*^ dw d^-*1*^); SLA, specific leaf area (cm^*2*^ g^-*1*^ dw); SRL, specific root length (cm g^-*1*^ dw); N net uptake capacity (nmol N g^-*1*^ fw h^-*1*^), ${NH}_{4}^{+}$, ammonium; ${NO}_{3}^{-}$, nitrate; Gln-N, glutamine; Arg-N, arginine; total soluble amino acid-N (mg amino acid-N g^-*1*^ dw); total soluble protein-N (mg protein-N g^-*1*^ dw). (a) Only at high soil N availability. (b) Only at low soil N availability. No letter: effect of competition regardless of soil N availability.*

#### Invasive Tree Species – Differences in the Response to Competitor Identity Regarding Total Biomass, Growth Indices, N Acquisition, and N Pools in the Leaves and Fine Roots

**FIGURE 2:**
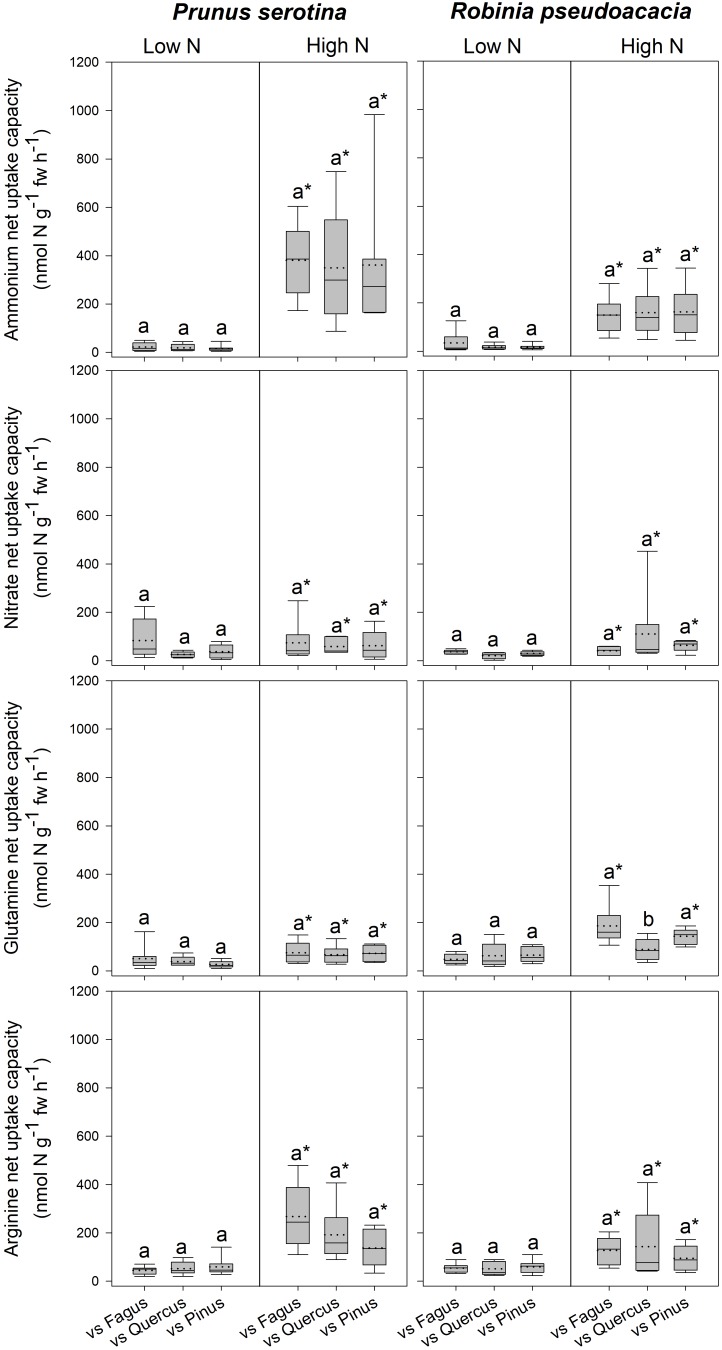
Ammonium, nitrate, glutamine-N, and arginine-N net uptake capacity (nmol N g^-1^ fw h^-1^) by fine roots of *Prunus serotina* and *Robinia pseudoacacia* seedlings at low and high soil N availability under different competition regimes. vs. Fagus = competition with *Fagus sylvatica*; vs. Quercus = competition with *Quercus robur*; vs. Pinus = competition with *Pinus sylvestris*. Box plots show mean (dotted line) and median (continuous line). Different letters indicate significant differences between competition regimes within a specific soil N availability treatment, and asterisks indicate significant differences between soil N availability treatments detected using permutational analysis of variance (*p* < 0.05).

Seedlings of *Prunus* had a significantly lower SLA when competing with *Fagus* than when competing with *Quercus* (Table 3 and Supplementary Table 7), while there were no differences between different competitor identities with respect to any of the other measured parameters (Figure 2, Table 3 and Supplementary Tables 7, 8A). Moreover, *Robinia* seedlings competing with *Fagus* had a significantly lower SLA (regardless of soil N availability), and higher total soluble amino acid-N content in the fine roots (only at low soil N availability) than seedlings competing with *Quercus* or *Pinus* (Table 3 and Supplementary Tables 7, 8A,B). Furthermore, seedlings of *Robinia* competing with *Quercus* had significantly lower glutamine-N net uptake capacity than those competing with either *Fagus* or *Pinus* (only at high soil N availability) (Figure 2). All other measured parameters did not differ significantly between different competitor identities for this species (Figure 2, Table 3 and Supplementary Tables 7, 8A).

### Effects of High Compared to Low Soil N Availability on Native and Invasive Tree Seedlings Growing in Competition

#### Native Tree Species – Effects of High Compared to Low Soil N Availability on Total Biomass, Growth Indices, N Acquisition, and N Pools in the Leaves and Fine Roots

For seedlings of *Fagus* a higher soil N availability resulted in significantly higher ammonium and arginine-N net uptake capacity (Figure 1), as well as a significantly lower SRL (Table 2B and Supplementary Table 7) and total soluble amino acid-N content in the leaves and fine roots (Table 2B and Supplementary Table 8A), regardless of competitor identity, while the rest of the measured parameters did not change significantly. For *Quercus*, ammonium and organic N net uptake capacity increased significantly (Figure 1), while RGR (Table 2B and Supplementary Table 7) and total soluble protein-N content in the fine roots (Table 2B and Supplementary Table 8A) decreased significantly at high compared to low soil N availability, regardless of competitor identity. Furthermore, higher soil N availability significantly reduced the total soluble amino acid-N content in the fine roots for *Quercus* seedlings when grown in competition with *Prunus* but not in seedlings grown in competition with *Robinia* (Table 2B and Supplementary Tables 8A,B). All other measured parameters remained unchanged between soil N availability treatments. For seedlings of *Pinus*, a high soil N availability significantly increased ammonium and arginine-N net uptake capacity (Figure 1), whereas RGR (Table 2B and Supplementary Table 7) and total soluble amino acid-N content in the leaves decreased (Table 2B and Supplementary Table 8A) compared to low soil N availability regardless of competitor identity, while the other measured parameters did not differ significantly.

#### Invasive Tree Species – Effects of High Compared to Low Soil N Availability on Total Biomass, Growth Indices, N Acquisition, and N Pools in the Leaves and Fine Roots

*Prunus* seedlings at high compared to low soil N availability significantly increased their ammonium and organic N net uptake capacity (Figure 2), and decreased SRL (Table 2B and Supplementary Table 7), total soluble amino acid-N contents in the leaves and fine roots, as well as total soluble protein-N content in the leaves (Table 2B and Supplementary Table 8A) regardless of the competing species. There were no effects of soil N availability on any other measured parameter. For seedlings of *Robinia*, higher soil N availability resulted in significant increases in inorganic N and arginine-N net uptake capacity (Figure 2), as well as significantly decreased total soluble amino acid-N content in the leaves and total soluble protein-N content in the fine roots regardless of competition (Table 2B and Supplementary Table 8A). However, glutamine-N net uptake capacity increased significantly with higher soil N availability in seedlings competing with *Fagus* and *Pinus*, but not when competing with *Quercus* (Figure 2). Similarly, total soluble amino acid-N content in the fine roots decreased significantly with higher soil N availability in seedlings grown in competition with *Fagus* and *Quercus*, but not when grown in competition with *Pinus* (Table 2B and Supplementary Tables 8A,B). Soil N availability did not affect other parameters measured in *Robinia*.

### N Acquisition Preferences of Native and Invasive Species for Specific N Sources at Low and High Soil N Availability

Comparing inorganic and organic N acquisition among N forms for each combination of species and competitor identity as well as for the single grown seedlings, we found general preference patterns at low and high soil N availability. At low soil N availability, organic N was preferred over inorganic N across species and competitor identities (Tables 4, 5), with a few exceptions that showed no preference for any N source: single seedlings of *Quercus* and *Prunus*, seedlings of *Quercus* competing with *Robinia*, and seedlings of *Prunus* and *Robinia* competing with *Fagus* (Tables 4, 5). At high soil N availability, a general pattern of preference for ammonium was found followed by organic N sources over nitrate. However, no preferences were found in single seedlings of *Prunus* and *Robinia*, seedlings of *Pinus* competing with *Robinia*, seedlings of *Robinia* competing with *Quercus*, and seedlings of *Prunus* and *Robinia* competing with *Pinus* (Tables 4, 5).

**Table 4:** Differences between ammonium (${NH}_{4}^{+}$), nitrate (${NO}_{3}^{-}$), glutamine-N (Gln-N), and arginine-N (Arg-N) net uptake capacity of the fine roots of *Fagus sylvatica*, *Quercus robur*, and *Pinus sylvestris* seedlings grown single and in competition at low and high soil N availability.

	Soil N availability	*Fagus*	*Quercus*	*Pinus*
Single grown seedlings	Low	Gln-N, Arg-N > ${NH}_{4}^{+}$	n.s.	Gln-N, Arg-N > ${NH}_{4}^{+}$
		Gln-N > ${NO}_{3}^{-}$		Gln-N > ${NO}_{3}^{-}$
	High	${NH}_{4}^{+}$, Arg-N > Gln-N > ${NO}_{3}^{-}$	n.s.	${NH}_{4}^{+}$, Gln-N, Arg-N > ${NO}_{3}^{-}$
Seedlings in competition with *Prunus*	Low	Gln-N, Arg-N > ${NO}_{3}^{-}$	Gln-N, Arg-N > ${NH}_{4}^{+}$	Gln-N, Arg-N > ${NH}_{4}^{+}$, ${NO}_{3}^{-}$
		Gln-N > ${NH}_{4}^{+}$
	High	${NH}_{4}^{+}$ > Arg-N > Gln-N > ${NO}_{3}^{-}$	${NH}_{4}^{+}$, Gln-N, Arg-N > ${NO}_{3}^{-}$	${NH}_{4}^{+}$, Arg-N > Gln-N > ${NO}_{3}^{-}$
Seedlings in competition with *Robinia*	Low	Gln-N, Arg-N > ${NH}_{4}^{+}$	n.s.	Gln-N, Arg-N > ${NH}_{4}^{+}$, ${NO}_{3}^{-}$
	High	${NH}_{4}^{+}$ > Gln-N > ${NO}_{3}^{-}$	${NH}_{4}^{+}$, Arg-N > ${NO}_{3}^{-}$, Gln-N	n.s.
		Arg-N > ${NO}_{3}^{-}$


*Only significant differences are presented. n.s., no significant differences between net uptake capacity of different N forms.*

**Table 5:** Differences between ammonium (${NH}_{4}^{+}$), nitrate (${NO}_{3}^{-}$), glutamine-N (Gln-N), and arginine-N (Arg-N) net uptake capacity of the fine roots of *Prunus serotina* and *Robinia pseudoacacia* seedlings grown single and in competition at low and high soil N availability.

	Soil N availability	*Prunus*	*Robinia*
Single grown seedlings	Low	n.s.	Gln-N, Arg-N > ${NH}_{4}^{+}$
	High	${NH}_{4}^{+}$, Arg-N > Gln-N > ${NO}_{3}^{-}$	n.s.
Seedlings in competition with *Fagus*	Low	n.s.	n.s.
	High	${NH}_{4}^{+}$, Arg-N > Gln-N, ${NO}_{3}^{-}$	${NH}_{4}^{+}$, Gln-N, Arg-N > ${NO}_{3}^{-}$
Seedlings in competition with *Quercus*	Low	Arg-N > ${NH}_{4}^{+}$, ${NO}_{3}^{-}$	Gln-N, Arg-N > ${NH}_{4}^{+}$
	High	${NH}_{4}^{+}$, Arg-N > ${NO}_{3}^{-}$, Gln-N	n.s.
Seedlings in competition with *Pinus*	Low	Arg-N > ${NH}_{4}^{+}$, Gln-N	Gln-N, Arg-N > ${NH}_{4}^{+}$, ${NO}_{3}^{-}$
	High	n.s.	n.s.


*Only significant differences are presented. n.s., no significant differences between net uptake capacity of different N forms.*

With regard to the specific preferences of each species across different competitor identities and single grown seedlings, native *Fagus* and *Pinus* had in general similar preferences for N sources regardless of competitor identity or whether they grow single or in competition (Table 4). However, native *Quercus* showed no preference for any N source either when grown single or in competition with *Robinia*, but preferred organic N over ammonium when grown in competition with *Prunus* (at low soil N availability). Moreover, at high soil N availability, the preferences of *Quercus* shifted from no preference when grown single to preferring ammonium and organic N over nitrate when grown in competition with *Prunus* or *Robinia* (Table 4). For the invasive species, *Prunus* had no preference of N sources when grown single but generally preferred arginine-N over glutamine-N and inorganic N when competing with *Quercus* or *Pinus* at low soil N availability, while preferences for N sources did not shift across competitor identities at high soil N availability (Table 5). Seedlings of *Robinia* preferred organic over inorganic N sources regardless of competitor identity as well as when grown single at low soil N availability, except for seedlings competing with *Fagus* which showed no preference. However, at high soil N availability, *Robinia* seedlings preferred ammonium and organic N over nitrate when competing with *Fagus*, while seedlings competing with other native species or grown single took up all N forms without preference (Table 5).

## Discussion

### Strategies of Native and Invasive Species Growing Without Competition

#### Invasive Species Succeed With Fast Growth, but Natives Could Counter With Preference of Organic N Acquisition

Comparing single grown invasive and native species, invasive species showed traits characteristic for fast-growing species. The invasive species had higher total biomass, RGR, and SRL, as well as total soluble amino acid-N and protein-N levels in both leaves and fine roots compared to the natives. A fast RGR is related to a rapid increase in biomass as well as high leaf nutrient concentrations, whereas slow RGR is related to a higher investment of resources in defense (Reich et al., 1997; Aerts, 1999). A higher SRL allows to forage larger soil volumes and is linked to a higher competitive ability for belowground resources (Aerts, 1999). Our results suggest a potential of the studied invasive species to outcompete the native ones via rapid growth and exploitation of above- and below-ground resources (Vilà and Weiner, 2004; Gioria and Osborne, 2014), which is in line with previous studies that found higher biomass (e.g., Lee et al., 2004; Closset-Kopp et al., 2007; Ding et al., 2012; Kawaletz et al., 2013), and monthly height increments (e.g., Ding et al., 2012) of *P. serotina* and *R. pseudoacacia* compared to plant species native to Europe and Asia. Moreover, the higher content of N metabolites in the fine roots is also linked to fast growing species compared to slow growers (e.g., *A. pseudoplatanus* compared to *F. sylvatica* in Li et al., 2015). For *Robinia*, a higher total N content in the leaves and fine roots compared to the other species is most likely associated with its N_2_-fixing ability (McKey, 1994; De Marco et al., 2013; Salpagarova et al., 2014). With regard to the N acquisition strategies, inorganic N acquisition was similar between invasive and native species. However, the lower organic N acquisition for the invasive compared to the native species suggests that native species could have a competitive advantage over the invasives via better exploitation of soil organic N. Although the invasives have faster growth, their species-specific N acquisition does not suggest a competitive advantage over natives.

#### With Higher Soil N Availability N Acquisition Strategies of Native and Invasive Seedlings Are Similar, Whereas N Allocation to N Pools in the Leaves and Fine Roots Is Species-Specific

In our study, the responses of single growing native and invasive species to high compared to low soil N availability were similar for all species for N acquisition, biomass and growth indices, whereas the allocation of N to N pools in the fine roots and leaves depended on the species. More specifically, inorganic and organic N acquisition increased with rising soil N levels, while biomass and growth indices showed no response, except for an increased root:shoot ratio for *Robinia* indicating a greater allocation to root biomass with increasing soil N supply. The allocation of N to N pools in the leaves and fine roots was species-specific with one of three responses with higher compared to lower soil N availability: (1) an increase in total soluble protein-N levels in the leaves (i.e., for native *Fagus*), (2) a decrease in total soluble amino acid-N levels in the leaves (i.e., for native *Quercus*), and (3) no change of total soluble amino acid-N or protein-N levels in neither leaves nor fine roots (i.e., for native *Pinus* and both invasives *Prunus* and *Robinia*).

The increase in total soluble protein-N levels in the leaves of *Fagus* with higher soil N availability in combination with no change in overall biomass suggests that N is stored as Rubisco for later mobilization (Millard, 1988; Masclaux-Daubresse et al., 2010). In contrast, *Quercus* had a reduced total soluble amino acid-N levels in the leaves which suggests that soluble amino acids are used to produce other compounds not quantified here, e.g., compounds related to defense. N allocation to N pools did not differ with increased soil N availability in *Pinus*, *Prunus*, and *Robinia* which is likely related to life history traits resulting in a relative independence of external soil N supply: *Pinus* is a conifer with needles present throughout the year in which N is stored (Houston et al., 2016b), *Robinia* can fix atmospheric N_2_ (Vítková et al., 2017), and *Prunus* allocates N to allelopathic compounds, such as cyanogenic glycosides (Csiszár, 2009), thereby potentially inhibiting N uptake by competitors.

Overall, for all studied species, the increased inorganic and organic N acquisition as well as the reduced levels of N metabolites in leaves and fine roots with rising soil N availability indicate plant physiological adjustments to meet the N demands with different soil N supply (BassiriRad, 2000). The lack of response with regard to biomass or growth indices suggests that the acquired N is either used for metabolism maintenance or assigned to storage (Millard and Grelet, 2010). Plant N acquisition is directly related to soil N supply, thus, higher organic and inorganic N acquisition with higher soil N availability reflects the active regulation of N uptake (Kulmatiski et al., 2017) which has already been reported for seedlings of *F. sylvatica* (e.g., Li et al., 2015) and *P. sylvestris* (e.g., Simon et al., 2013).

### Native Species Respond to Competition With Invasives With Distinct Species-Specific Strategies

The native species showed distinct species- and also parameter-specific strategies in their responses to competition with different invasive species that were partly mediated by soil N availability. When competing with *Prunus* compared to *Robinia*: (1) *Fagus* had lower RGR and N acquisition without changes in biomass or N metabolite levels, (2) *Quercus* had lower biomass and total soluble amino acid-N levels without changes in N acquisition, whereas (3) *Pinus* only had higher SRL, without changes in biomass, N acquisition or N metabolite levels. More specifically, the lower RGR combined with N acquisition in *Fagus* in competition with *Prunus* compared to *Robinia* suggests that the response strength to competition with an invasive species depends on the competitor. The slower growth combined with a reduced N acquisition from the soil indicates a remobilization of internal N in compensation for the impaired N acquisition from the soil assuming a similar N demand. This strategy of a reduced growth rate (Li et al., 2015) and N acquisition (Simon et al., 2010, 2014) was found also in response to competition with fast-growing *A. pseudoplatanus*, thus indicating a general response of *F. sylvatica* to fast-growing competitors. In contrast, *Quercus* had a decreased biomass as well as total soluble amino acid-N content in the fine roots (only at high soil N availability) in competition with *Prunus* compared to *Robinia*, with no change in N acquisition suggesting a shift in the allocation of N from growth to storage and/or the synthesis of defense compounds depending on the competing species (Millard and Grelet, 2010). The lower total soluble amino acid-N levels in the fine roots when competing with *Prunus* compared to *Robinia* but only a high soil N availability indicates a mediation of the competitive response by soil N supply. Lower amino acid-N levels in the fine roots have been related to slower growth (Simon et al., 2010), whereas an increase in total soluble amino acid levels in the fine roots as a result of competition (Li et al., 2015) was found in fast-growing sycamore maple (*A. pseudoplatanus* L.). However, *Fagus* showed a different response, despite also being a slow growing species which indicates that other species-specific factors regulate the response to competition in terms of total soluble amino acid-N content in the fine roots that were not considered in our study. For seedlings of *Pinus* the sole response to competition with *Prunus* compared to *Robinia* was a higher SRL which regulates the access to N (Andersen et al., 2017). Growing longer, thinner roots allows *Pinus* seedlings to maximize resource capture without changing N acquisition capacity per root, thereby being able to tolerate competition with no effects on productivity. Unlike both deciduous species *Fagus* and *Quercus*, coniferous *Pinus* did not show different responses to different invasive competitors regarding N acquisition and N pools in leaves and fine roots, likely because conifers maintain needles in which N is stored throughout the year (Millard and Grelet, 2010) and can be utilized when needed. By drawing on these resources, *Pinus* can buffer the different competitive effects of the invasive species. Noticeably, none of our native study species responded differently depending on the competitor with regard to their total soluble protein-N levels in the leaves or fine roots. Previous studies on temperate tree seedlings including *F. sylvatica* (Simon et al., 2010, 2014; Li et al., 2015) found changes in total soluble protein-N contents in the fine roots when grown in intraspecific or interspecific competition indicating *de novo* protein synthesis as an adaptive response to competition. In the present study, we compare native species responses to different invasive species rather than intraspecific vs. interspecific competition within native species. Thus, it is possible that protein-N levels changed for the single species when grown in competition, but the response was similar with different competitors.

Overall, the response of native seedlings in competition with invasive species is species-specific and reflects different coping mechanisms related to the species life history and growth strategies. For instance, coniferous species such as *Pinus* maintain their needles throughout the year resulting in higher N storage capacities compared to deciduous species like *Fagus* and *Quercus* (Millard and Grelet, 2010). As a consequence, conifers might be less responsive to the different competitive effects of the invasive species in terms of the competition for N. All three native species responded stronger when competing with *Prunus* than *Robinia*.

#### Why Is *P. serotina* a Stronger Competitor Than *R. pseudoacacia* for the Studied Native Species?

In our study, the native tree seedlings competing with *Prunus* responded with decreases in N acquisition, total biomass, RGR, and total soluble amino acid-N levels in the fine roots than when competing with *Robinia* which can be explained by its relatively high root biomass, and, in turn, a better exploitation of belowground resources of *Prunus* (Casper and Jackson, 1997; Kawaletz et al., 2014) compared to all other species in our study which is linked to increased success rates of invasive plants (e.g., Grotkopp and Rejmánek, 2007; Robakowski and Bielinis, 2011; Kawaletz et al., 2013; Gioria and Osborne, 2014). Moreover, *Prunus* produces cyanogenic compounds also in the roots, a strategy that might have had an allelopathic effect on the neighboring plants (Csiszár, 2009; Robakowski et al., 2016) and contributed to the general negative influence of *Prunus* on the native species in our study. Overall, the higher competitive ability of *Prunus* in our study is in accordance with earlier work by others; however, previous studies investigated the effects of *Prunus* and *Robinia* when competing with native species mainly considering plant growth and biomass (e.g., Closset-Kopp et al., 2011; Robakowski and Bielinis, 2011; Kawaletz et al., 2013, 2014), whereas we found not only competition effects on growth and biomass but also on N acquisition and N allocation to different N pools depending on the species.

### Invasive *R. pseudoacacia* Reacts Stronger to Competition With Native Seedlings Than *P. serotina*

The invasive species used in our study responded to competition depending on the native competitor species and soil N availability: the response of *Prunus* to competition with native seedlings was an increase in SLA when grown in competition with *Quercus* compared to competition with *Fagus*, but not *Pinus*. Usually, plants with a high SLA have a lower competitive effect related to a shorter leaf lifespan resulting in a lower leaf mass fraction (Kuster et al., 2016); which is, however mostly related to limiting resource conditions (Knops and Reinhart, 2000). In contrast, having a higher SLA when belowground resources are sufficient becomes a competitive advantage as a strategy of fast resource acquisition (Liu et al., 2017). Therefore, given the higher root biomass of *Prunus* compared to our native study species (see section “Invasive Species Succeed With Fast Growth, But Natives Could Counter With Preference of Organic N Acquisition”), *Prunus* was a strong competitor for belowground resources, and enhanced its competitive ability and fast growth strategy by increasing light interception via a higher SLA (Liu et al., 2017).

In contrast, seedlings of *Robinia* responded to competition depending on the native species, with a decrease in organic N acquisition in competition with *Quercus* compared to *Pinus* and *Fagus*, as well as an increased SLA combined with a decrease in fine root total soluble amino acid-N content in competition with *Quercus* and *Pinus* compared to *Fagus*. Notably, the decrease in fine root total soluble amino acid-N content with competition was found only at low soil N availability, the decrease in organic N acquisition only at high soil N availability. This response dependency on soil N availability for *Robinia* but not *Prunus* indicates that *Robinia* shows a strong response to the combined effects of competition and soil N availability, whereas the response of *Prunus* is not mediated by soil N supply. The increase in SLA when competing with *Quercus* compared to *Fagus* was found regardless of soil N availability and suggests a potential reduced competitive ability of *Robinia* competing with *Quercus*, especially in combination with the reduced organic N acquisition and total soluble amino acid-N content in the fine roots indicating an impaired N nutrition. In general, leaves with a high SLA represent a disadvantage for plant growth under limiting nutrient conditions, because of the negative correlation with leaf lifespan, and therefore increased N losses through leaf senescence (Reich et al., 1997; Knops and Reinhart, 2000).

Overall, the investigated invasive species responded at different levels (i.e., morphological and physiological) depending on the identity of the native competitor suggesting that *Robinia* represents a lesser threat than *Prunus* at the investigated time scale. However, the impact of *Robinia* might become more important in the long run as a result of its capacity to fix atmospheric N_2_ and therefore potentially alter biogeochemical processes in invaded ecosystem (Strayer et al., 2006; Medina-Villar et al., 2016). By modifying soil N availability, for example, N_2_-fixing plants might influence community composition and diversity (i.e., by favoring the establishment of nitrophilous species) (Staska et al., 2014), and consequently impact on plant–plant interactions such as competition.

### Higher Soil N Availability Results in Increased N Acquisition but No Change in Biomass or Growth Indices for All Species in Competition, While a Reduction in N Pool Levels Is Species- and Organ-Specific

Seedlings of native and invasive species growing in competition in our study varied their responses with high soil N supply. Although, none of them increased in biomass with high soil N supply seedlings of *Fagus* and *Prunus* had lower SRL indicating that foraging for nutrients via an investment in longer, thinner roots is only required when soil nutrients are limiting (Zhu et al., 2016). In all species, organic and inorganic N uptake increased with high compared to low soil N supply which is a key driver of N acquisition in woody species (Simon et al., 2017) as reported for *F. sylvatica* (Stoelken et al., 2010; Li et al., 2015), *P. sylvestris* (Simon et al., 2013), and *A. pseudoplatanus* (Li et al., 2015). However, reduced total soluble amino acid-N and total soluble protein-N levels with higher soil N availability were species- as well as organ-specific in our study. In general, plants have to rely less on internal N storage to maintain growth and metabolic functions when N is readily available in the soil (Millard and Grelet, 2010). For *Fagus*, our results are in contrast with a previous study that found no changes in total soluble amino acid-N contents in the fine roots with increasing soil N availability neither in intraspecific competition nor in competition with *A. pseudoplatanus* (Li et al., 2015). However, in our study, the response of native species to competition with invasive species in terms of N strategies is evaluated for the first time and suggests a specific response of native species to combined soil N availability and competition with the invasive species *Prunus* and *Robinia*.

Species responded to soil N availability mostly regardless of competitor identity, however, for some species an interaction between soil N availability and competitor was detected. For example, the reduction in total soluble amino acid-N levels in the fine roots with at high compared to low soil N availability described above was found in some species only with specific competitors (i.e., for *Quercus* when competing with *Prunus*, and for *Robinia* when competing with *Fagus* or *Quercus*). Specifically, for *Quercus* this suggests that invasive species *Prunus* induces a stronger response than invasive *Robinia*, a result that is consistent with the general responses of *Quercus* to *Prunus* described in Section “Native Species Respond to Competition With Invasives With Distinct Species-Specific Strategies.” We found no effects of competition on seedling total soluble amino acid-N or total soluble protein-N levels in the leaves which contrasts with studies reporting the invasion by *Prunus* to affect foliar N levels of native mature tree species including the ones considered here (e.g., Aerts et al., 2017). This suggests an effect of plant age on the competition response, as well as the possibility that soil N availability in our study was a strong driver of foliar N levels, thereby overriding the potential effects of competition.

### Native and Invasive Species Share Common Preferences for N Sources Regardless of Soil N Availability, but There Are Species-Specific Patterns Between Competition Regimes and Competitor Identities

The common N preference patterns across species in our study depended on soil N availability and species strategies in response to competition. At low soil N availability, organic N was taken up preferentially over inorganic N by seedlings of both native and invasive suggesting that tree seedlings maintain their metabolism and growth by drawing upon a wider variety of N sources. However, at high soil N availability, ammonium-N was the preferred N form followed by organic N, while nitrate-N was the least preferred N form taken up by tree seedlings, as also found for *F. sylvatica* (Stoelken et al., 2010) and *P. sylvestris* (Simon et al., 2010). Nitrate acquisition from the soil is inhibited by high concentrations of amino acids (particularly glutamine) and ammonium (Näsholm et al., 2009; Stoelken et al., 2010). Both native and invasive species preferred specific N sources linked to other aspects considered in our study. For example, *Quercus* preferred organic N forms over ammonium only when grown in competition with invasive *Prunus*. *Pinus* did not shift N source preferences. Both invasive species also lacked a preference for certain N forms when competing with *Fagus* (at low soil N availability) or *Pinus* (at high soil N availability) which corresponds with their general stronger reaction to competition with *Quercus* (see section “Invasive *R. pseudoacacia* Reacts Stronger to Competition With Native Seedlings Than *P. serotina*”). Overall, N form preferences matched the general response strategies of native and invasive species to differing competitors that were found for growth, N acquisition and allocation of N to N pools.

## Conclusion

In our study, we found that invasive species display traits that grant them competitive advantage, such as fast growth rates, which however did not result in a generally higher N acquisition of invasive species, because native species had a higher organic N net uptake capacity that would allow them to better utilize soil N sources, and thus potentially avoid competition with neighboring invasive species. When growing in competition, native tree seedlings showed a stronger response to competition with *Prunus* than *Robinia*, although the response variable changed between native species indicating the use of species-specific strategies of native seedlings to cope with the competition with invasive plants. These are further mediated by soil N availability in some cases. These strategies reflected the differences between native species in terms of life history and growth traits. The stronger response to *Prunus* could be related to the higher total biomass of this species and its ability to produce allelopathic compounds. Furthermore, when comparing the responses of invasive species, *Robinia* responded to different native competitors at different morphological and physiological levels, further influenced by soil N availability, while *Prunus* only showed changes in SLA, without interaction with soil N availability. This suggests that *Prunus* might have a stronger competitive advantage over native species than *Robinia* at least at the time scale of our study. Moreover, considering that native *Quercus* responded more negative to competition with *Prunus* than *Robinia*, as well as the several responses of *Robinia* to competition with *Quercus*, our results suggest that specifically *Quercus* could have a competitive advantage over invasive *Robinia*, but not over invasive *Prunus*. However, it is possible that the impact of *Robinia* becomes more important in the longer run at the plant community level, as a result of its capacity to fix atmospheric N_2_ and therefore potentially alter biogeochemical processes in an invaded ecosystem. With regard to the effects of high compared to low soil N availability on competing seedlings, we found common patterns among species similar to those displayed by single grown seedlings regarding N acquisition (increased with soil N availability), but not regarding N allocation, i.e., in single seedlings N allocation was scarcely influenced by soil N availability, while in competing seedlings there was a general decrease in total soluble amino acid-N levels for most species, which suggests stronger responses to increased soil N availability when in combination with competition. Generally, our results highlight *Prunus* as a potential greater threat to seedlings of native species than *Robinia* in the time frame considered here. Overall, our results provide novel insights into the different species-specific effects of invasive species on native seedlings not only with regard to growth parameters, but also underlying physiological processes such as N acquisition and internal allocation. However, our study included only five species (three natives, two invasives) differing in their ecological background, with a focus on the competition for N rather than other resources, such as light and/or space, thus more detailed studies are still required in the future. Furthermore, the results should be validated in long term studies conducted in the field. In conclusion, the species-specific nature of competition between native and invasive tree seedlings should be considered in forest management strategies in the future.

## Data Availability

The raw data supporting the conclusions of this manuscript will be made available by the authors, without undue reservation, to any qualified researcher.

## Author Contributions

AB and JS conceived the experimental design and manuscript writing. AB conducted the ^15^N uptake experiments and evaluated the data. KP contributed stable isotope analyses and ideas to data interpretation. All authors agreed on the final version of the manuscript.

## Conflict of Interest Statement

The authors declare that the research was conducted in the absence of any commercial or financial relationships that could be construed as a potential conflict of interest.
