# Supplementary material for: Species-Specific Outcome in the Competition for Nitrogen Between Invasive and Native Tree Seedlings
Source: Front Plant Sci. 2019 Mar 29;10:337. doi: 10.3389/fpls.2019.00337 (PMC6449475; doi:10.3389/fpls.2019.00337)
Supplement: Supplementary file 1 [file Data_Sheet_1.docx]

Supplementary Material

Species-specific outcome in the competition for nitrogen between invasive and native tree seedlings

Andrea Bueno*, Karin Pritsch, Judy Simon

*** Correspondence:** Andrea Bueno: andrea.bueno@uni-konstanz.de

**Supplementary Table 1.** PERMANOVA results of the differences between species and the effect of high compared to low soil N availability on total biomass (g), root:shoot ratio, relative growth rate (RGR, g dw g^-1^ dw d^-1^), specific leaf area (SLA, cm^2^ g^-1^ dw), and specific root length (SRL, cm g^-1^ dw) of *Fagus sylvatica*, *Quercus robur*, *Pinus sylvestris*, *Prunus serotina*, and *Robinia pseudoacacia* single grown seedlings. SLA was not measured for *P. sylvestris*. Pairwise comparisons were performed only when a significant difference was detected in the main test. Significant values (p ≤ 0.05) are indicated in bold.

|  | **Total biomass** | | **Root:shoot** | | **RGR** | | **SLA** | | **SRL** | |
| --- | --- | --- | --- | --- | --- | --- | --- | --- | --- | --- |
| ***Main test*** | ***Pseudo-F*** | ***P(perm)*** | ***Pseudo-F*** | ***P(perm)*** | ***Pseudo-F*** | ***P(perm)*** | ***Pseudo-F*** | ***P(perm)*** | ***Pseudo-F*** | ***P(perm)*** |
| Soil N availability | 0.065 | 0.794 | 0.095 | 0.753 | 0.048 | 0.829 | 1.263 | 0.265 | 8.497 | **0.005** |
| Species | 44.54 | **<0.001** | 122.83 | **<0.001** | 23.767 | **<0.001** | 32.559 | **<0.001** | 17.518 | **<0.001** |
| Soil N availability x Species | 1.922 | 0.1173 | 1.146 | 0.345 | 0.658 | 0.622 | 0.282 | 0.842 | 1.123 | 0.362 |
| ***Pairwise comparisons*** | ***t*** | ***P(perm)*** | ***t*** | ***P(perm)*** | ***t*** | ***P(perm)*** | ***t*** | ***P(perm)*** | ***t*** | ***P(perm)*** |
| *Fagus* vs. *Prunus* | 10.105 | **<0.001** | 5.089 | **<0.001** | 6.023 | **<0.001** | 8.897 | **<0.001** | 0.967 | 0.361 |
| *Quercus* vs. *Prunus* | 7.229 | **<0.001** | 7.024 | **<0.001** | 6.159 | **<0.001** | 5.165 | **<0.001** | 2.108 | 0.052 |
| *Pinus* vs. *Prunus* | 8.671 | **<0.001** | 19.831 | **<0.001** | 5.120 | **<0.001** | - | - | 1.082 | 0.294 |
| *Fagus* vs. *Robinia* | 6.823 | **<0.001** | 13.785 | **<0.001** | 6.801 | **<0.001** | 1.619 | 0.115 | 5.144 | **<0.001** |
| *Quercus* vs. *Robinia* | 3.435 | **0.001** | 13.449 | **<0.001** | 5.914 | **<0.001** | 4.788 | **<0.001** | 3.910 | **0.002** |
| *Pinus* vs. *Robinia* | 5.835 | **<0.001** | 3.456 | **0.001** | 5.676 | **<0.001** | - | - | 5.863 | **<0.001** |
| *Fagus* vs. *Quercus* | 4.323 | **<0.001** | 2.515 | **0.014** | 3.278 | **0.002** | 4.203 | **<0.001** | 0.936 | 0.353 |
| *Fagus* vs. *Pinus* | 0.309 | 0.765 | 14.878 | **<0.001** | 0.651 | 0.523 | - | - | 0.287 | 0.782 |
| *Quercus* vs. *Pinus* | 3.733 | **<0.001** | 14.185 | **<0.001** | 1.752 | 0.096 | - | - | 1.405 | 0.176 |
| *Prunus* vs. *Robinia* | 3.427 | **0.002** | 17.737 | **<0.001** | 1.709 | 0.095 | 8.042 | **<0.001** | 6.524 | **<0.001** |

**Supplementary Table 2.** PERMANOVA results of the differences between species and the effect of high compared to low soil N availability on total soluble amino acid-N (mg amino acid-N g^-1^ dw) and protein-N (mg protein-N g^-1^ dw) levels in leaves and fine roots of *Fagus sylvatica*, *Quercus robur*, *Pinus sylvestris*, *Prunus serotina*, and *Robinia pseudoacacia* single grown seedlings. Pairwise comparisons were performed only when a significant difference was detected in the main test. Significant values (p ≤ 0.05) are indicated in bold.

|  | **Leaves** | | | | **Fine roots** | | | |
| --- | --- | --- | --- | --- | --- | --- | --- | --- |
|  | **Total soluble amino acid-N** | | **Total soluble protein-N** | | **Total soluble amino acid-N** | | **Total soluble protein-N** | |
| ***Main test*** | ***Pseudo-F*** | ***P(perm)*** | ***Pseudo-F*** | ***P(perm)*** | ***Pseudo-F*** | ***P(perm)*** | ***Pseudo-F*** | ***P(perm)*** |
| Soil N availability | 10.605 | **0.0012** | 5.0134E-2 | 0.82 | 2.7574 | 0.1074 | 0.12312 | 0.7309 |
| Species | 27.046 | **0.0001** | 5.271 | **0.0009** | 5.0442 | **0.0026** | 6.465 | **0.0005** |
| Soil N availability x Species | 1.1957 | 0.3314 | 2.353 | 0.0689 | 2.1567 | 0.0864 | 2.0475 | 0.1037 |
| ***Pairwise comparisons*** | ***t*** | ***P(perm)*** | ***t*** | ***P(perm)*** | ***t*** | ***P(perm)*** | ***t*** | ***P(perm)*** |
| *Fagus* vs. *Prunus* | 3.576 | **0.002** | 2.560 | **0.013** | 2.087 | 0.051 | 1.693 | 0.087 |
| *Quercus* vs. *Prunus* | 4.405 | **0.001** | 1.992 | 0.056 | 2.755 | **0.012** | 0.183 | 0.872 |
| *Pinus* vs. *Prunus* | 3.199 | **0.006** | 4.221 | **0.001** | 3.997 | **<0.001** | 0.464 | 0.663 |
| *Fagus* vs. *Robinia* | 6.462 | **<0.001** | 1.881 | 0.074 | 1.569 | 0.127 | 4.963 | **<0.001** |
| *Quercus* vs. *Robinia* | 6.713 | **<0.001** | 2.005 | 0.055 | 2.207 | **0.040** | 3.154 | **0.005** |
| *Pinus* vs. *Robinia* | 6.468 | **<0.001** | 0.578 | 0.581 | 3.414 | **0.004** | 2.447 | **0.022** |
| *Fagus* vs. *Quercus* | 0.189 | 0.856 | 0.362 | 0.725 | 0.423 | 0.685 | 2.240 | **0.039** |
| *Fagus* vs. *Pinus* | 0.666 | 0.516 | 1.776 | 0.099 | 1.437 | 0.196 | 2.466 | **0.017** |
| *Quercus* vs. *Pinus* | 1.035 | 0.323 | 1.999 | 0.060 | 1.504 | 0.149 | 0.722 | 0.488 |
| *Prunus* vs. *Robinia* | 4.381 | **<0.001** | 3.580 | **0.002** | 0.558 | 0.578 | 2.775 | 0.014 |

**Supplementary Table 3.** PERMANOVA results of the differences between species and the effect of high compared to low soil N availability on the ammonium (NH_4_^+^), nitrate (NO_3_^-^), glutamine-N (Gln-N), and arginine-N (Arg-N) net uptake capacity (nmol N g^-1^ fw h^-1^) of fine roots of *Fagus sylvatica*, *Quercus robur*, *Pinus sylvestris*, *Prunus serotina*, and *Robinia pseudoacacia* single grown seedlings. Pairwise comparisons were performed only when a significant difference was detected in the main test. Significant values (p ≤ 0.05) are indicated in bold.

|  | **NH_4_^+^** | | **NO_3_^-^** | | **Gln-N** | | **Arg-N** | | | |
| --- | --- | --- | --- | --- | --- | --- | --- | --- | --- | --- |
| ***Main test*** | ***Pseudo-F*** | ***P(perm)*** | ***Pseudo-F*** | ***P(perm)*** | ***Pseudo-F*** | ***P(perm)*** | ***Pseudo-F*** | | ***P(perm)*** | |
| Soil N availability | 61.374 | **<0.001** | 3.754 | **0.029** | 22.543 | **<0.001** | 62.626 | | <0.001 | |
| Species | 2.052 | 0.098 | 1.361 | 0.219 | 9.868 | **<0.001** | 5.772 | | <0.001 | |
| Soil N availability x Species | 1.374 | 0.259 | 1.306 | 0.260 | 1.233 | 0.306 | 2.907 | | **0.028** | |
| ***Pairwise comparisons*** | ***t*** | ***P(perm)*** | ***t*** | ***P(perm)*** | ***t*** | ***P(perm)*** | ***t*** | ***P(perm) (low N)*** | ***t*** | ***P(perm) (high N)*** |
| *Fagus* vs. *Prunus* | *Not applicable* | | *Not applicable* | | 5.710 | **<0.001** | 2.194 | **0.045** | 3.474 | **0.001** |
| *Quercus* vs. *Prunus* |  |  |  |  | 2.775 | **0.006** | 1.227 | 0.261 | 2.369 | **0.030** |
| *Pinus* vs. *Prunus* |  |  |  |  | 4.172 | **<0.001** | 2.356 | **0.046** | 2.403 | **0.037** |
| *Fagus* vs. *Robinia* |  |  |  |  | 4.861 | **<0.001** | 1.721 | 0.117 | 3.803 | **0.001** |
| *Quercus* vs. *Robinia* |  |  |  |  | 1.879 | 0.074 | 0.777 | 0.500 | 2.596 | **0.016** |
| *Pinus* vs. *Robinia* |  |  |  |  | 3.471 | **0.002** | 1.859 | 0.109 | 2.668 | **0.029** |
| *Fagus* vs. *Quercus* |  |  |  |  | 2.548 | **0.019** | 0.865 | 0.393 | 0.288 | 0.742 |
| *Fagus* vs. *Pinus* |  |  |  |  | 0.521 | 0.598 | 0.076 | 0.944 | 0.491 | 0.627 |
| *Quercus* vs. *Pinus* |  |  |  |  | 1.734 | 0.096 | 0.943 | 0.380 | 0.147 | 0.886 |
| *Prunus* vs. *Robinia* |  |  |  |  | 1.613 | 0.128 | 0.562 | 0.587 | 0.656 | 0.519 |

**Supplementary Table 4.** Mann-Whitney U-test results of the effect of high compared to low soil N availability on total biomass (g), root:shoot ratio, relative growth rate (RGR, g dw g^-1^ dw d^-1^), specific leaf area (SLA, cm^2^ g^-1^ dw), and specific root length (SRL, cm g^-1^ dw) of *Fagus sylvatica*, *Quercus robur*, *Pinus sylvestris*, *Prunus serotina*, and *Robinia pseudoacacia* single grown seedlings. SLA was not measured for *P. sylvestris*. Significant values (p ≤ 0.05) are indicated in bold.

|  | **Total biomass** | | **Root:shoot ratio** | | **RGR** | | **SLA** | | **SRL** | |
| --- | --- | --- | --- | --- | --- | --- | --- | --- | --- | --- |
|  | ***Mann–Whitney U*** | ***P*** | ***Mann–Whitney U*** | ***P*** | ***Mann–Whitney U*** | ***P*** | ***Mann–Whitney U*** | ***P*** | ***Mann–Whitney U*** | ***P*** |
| ***Fagus*** | 53.0 | 0.297 | 51.0 | 0.247 | 51.0 | 0.372 | 41.0 | 0.132 | 11.0 | 0.310 |
| ***Quercus*** | 54.0 | 0.201 | 52.0 | 0.165 | 3.0 | 0.800 | 68.0 | 0.605 | 4.0 | 0.114 |
| ***Pinus*** | 38.0 | 0.894 | 24.0 | 0.168 | 33.0 | 0.884 | - | - | 13.0 | 0.792 |
| ***Prunus*** | 49.0 | 0.121 | 60.0 | 0.341 | 54.0 | 0.201 | 58.0 | 0.289 | 12.0 | 0.662 |
| ***Robinia*** | 48.0 | 0.910 | 23.0 | **0.045** | 47.0 | 0.850 | 46.0 | 0.791 | 8.0 | 0.132 |

**Supplementary Table 5.** Mann-Whitney U-test results of the effect of high compared to low soil N availability on ammonium (NH_4_^+^), nitrate (NO_3_^-^), glutamine-N (Gln-N), and arginine-N (Arg-N) net uptake capacity (nmol N g^-1^ fw h^-1^) of fine roots of *Fagus sylvatica*, *Quercus robur*, *Pinus sylvestris*, *Prunus serotina*, and *Robinia pseudoacacia* single grown seedlings. Significant values (p ≤ 0.05) are indicated in bold.

|  | **NH_4_^+^** | | **NO_3_^-^** | | **Gln-N** | | **Arg-N** | |
| --- | --- | --- | --- | --- | --- | --- | --- | --- |
|  | ***Mann–Whitney U*** | ***P*** | ***Mann–Whitney U*** | ***P*** | ***Mann–Whitney U*** | ***P*** | ***Mann–Whitney U*** | ***P*** |
| ***Fagus*** | 0.0 | **0.001** | 6.0 | **0.035** | 10.0 | 0.138 | 0.0 | **<0.001** |
| ***Quercus*** | 1.0 | **0.004** | 21.0 | 0.279 | 9.0 | 0.053 | 0.0 | **0.004** |
| ***Pinus*** | 0.0 | **0.004** | 2.0 | **0.032** | 6.0 | **0.035** | 2.0 | **0.005** |
| ***Prunus*** | 0.0 | **0.004** | 9.0 | 0.180 | 7.0 | 0.051 | 1.0 | **0.002** |
| ***Robinia*** | 0.0 | **0.001** | 10.0 | 0.138 | 9.0 | 0.101 | 2.0 | **0.017** |

**Supplementary Table 6.** Mann-Whitney U-test results of the effect of high compared to low soil N availability on total soluble amino acid-N (mg amino acid-N g^-1^ dw) and protein-N (mg protein-N g^-1^ dw) levels in leaves and fine roots of *Fagus sylvatica*, *Quercus robur*, *Pinus sylvestris*, *Prunus serotina*, and *Robinia pseudoacacia* single grown seedlings. Significant values (p ≤ 0.05) are indicated in bold.

|  | **Leaves** | | | | **Fine roots** | | | |
| --- | --- | --- | --- | --- | --- | --- | --- | --- |
|  | **Total soluble amino acid-N** | | **Total soluble protein-N** | | **Total soluble amino acid-N** | | **Total soluble protein-N** | |
|  | ***Mann–Whitney U*** | ***P*** | ***Mann–Whitney U*** | ***P*** | ***Mann–Whitney U*** | ***P*** | ***Mann–Whitney U*** | ***P*** |
| ***Fagus*** | 14.0 | 0.931 | 2.0 | **0.017** | 7.0 | 0.177 | 10.0 | 0.429 |
| ***Quercus*** | 0.0 | **0.010** | 6.0 | 0.257 | 8.0 | 0.476 | 11.0 | 0.914 |
| ***Pinus*** | 7.0 | 0.093 | 15.0 | 0.699 | 11.0 | 0.310 | 18.0 | 1.000 |
| ***Prunus*** | 6.0 | 0.065 | 6.0 | 0.065 | 7.0 | 0.093 | 10.0 | 0.240 |
| ***Robinia*** | 7.0 | 0.177 | 11.0 | 0.310 | 13.0 | 0.485 | 6.0 | 0.065 |

**Supplementary Table 7.** PERMANOVA results of the effects of high compared to low soil N availability and competitor identity on total biomass (g), root:shoot ratio, relative growth rate (RGR, g dw g^-1^ dw d^-1^), specific leaf area (SLA, cm^2^ g^-1^ dw), and specific root length (SRL, cm g^-1^ dw) of *Fagus sylvatica*, *Quercus robur*, *Pinus sylvestris*, *Prunus serotina*, and *Robinia pseudoacacia* seedlings grown in competition. SLA was not measured for *P. sylvestris*. Significant values (p ≤ 0.05) are indicated in bold.

|  | **Total biomass** | | **Root:shoot ratio** | | **RGR** | | **SLA** | | **SRL** | |
| --- | --- | --- | --- | --- | --- | --- | --- | --- | --- | --- |
|  | ***Pseudo-F*** | ***P (perm)*** | ***Pseudo-F*** | ***P (perm)*** | ***Pseudo-F*** | ***P (perm)*** | ***Pseudo-F*** | ***P (perm)*** | ***Pseudo-F*** | ***P (perm)*** |
| ***Fagus*** | | | | | | | | | | |
| Soil N availability | 2.530 | 0.123 | 1.878 | 0.178 | 0.799 | 0.377 | 0.663 | 0.419 | 12.226 | **0.004** |
| Competitor identity | 1.663 | 0.206 | 0.128 | 0.729 | 6.239 | **0.019** | 0.035 | 0.851 | 3.744 | 0.073 |
| Soil N availability x Competitor identity | 0.094 | 0.771 | 0.099 | 0.747 | 0.037 | 0.847 | 1.784 | 0.199 | 3.865 | 0.064 |
| ***Quercus*** | | | | | | | | | | |
| Soil N availability | 1.511 | 0.244 | 0.025 | 0.890 | 4.138 | **0.049** | 0.127 | 0.723 | 0.123 | 0.729 |
| Competitor identity | 4.929 | **0.017** | 0.300 | 0.579 | 1.638 | 0.214 | 0.407 | 0.532 | 2.188 | 0.153 |
| Soil N availability x Competitor identity | 2.427 | 0.124 | 0.149 | 0.710 | 3.106 | 0.092 | 0.219 | 0.637 | 1.067 | 0.309 |
| ***Pinus*** | | | | | | | | | | |
| Soil N availability | 0.628 | 0.446 | 0.484 | 0.522 | 5.325 | **0.037** | - | - | 0.469 | 0.501 |
| Competitor identity | 0.743 | 0.397 | 0.261 | 0.633 | 1.891 | 0.190 | - | - | 5.756 | **0.026** |
| Soil N availability x Competitor identity | 2.598 | 0.115 | 1.751 | 0.198 | 0.663 | 0.419 | - | - | 0.006 | 0.941 |
| ***Prunus*** | | | | | | | | | | |
| Soil N availability | 0.189 | 0.657 | 0.813 | 0.589 | 2.610 | 0.119 | <0.001 | 0.986 | 5.403 | **0.025** |
| Competitor identity | 0.659 | 0.528 | 0.855 | 0.644 | 3.221 | 0.053 | 3.299 | **0.045** | 1.198 | 0.312 |
| Soil N availability x Competitor identity | 0.435 | 0.649 | 0.855 | 0.635 | 0.218 | 0.804 | 0.440 | 0.641 | 0.345 | 0.713 |
| ***Robinia*** | | | | | | | | | | |
| Soil N availability | 1.718 | 0.193 | 0.077 | 0.782 | 0.323 | 0.570 | 1.287 | 0.264 | 2.775 | 0.101 |
| Competitor identity | 1.132 | 0.340 | 0.366 | 0.691 | 0.672 | 0.512 | 5.915 | **0.006** | 0.632 | 0.542 |
| Soil N availability x Competitor identity | 0.237 | 0.790 | 0.005 | 0.994 | 0.195 | 0.831 | 1.984 | 0.149 | 0.007 | 0.993 |

**Supplementary Table 8A.** PERMANOVA results of the effects of high compared to low soil N availability and competitor identity on total soluble amino acid-N (mg amino acid-N g^-1^ dw) and protein-N (mg protein-N g^-1^ dw) levels in leaves and fine roots of *Fagus sylvatica*, *Quercus robur*, *Pinus sylvestris*, *Prunus serotina*, and *Robinia pseudoacacia* seedlings grown in competition. Significant values (p ≤ 0.05) are indicated in bold.

|  | **Leaves** | | | | **Fine roots** | | | |
| --- | --- | --- | --- | --- | --- | --- | --- | --- |
|  | **Total soluble amino acid-N** | | **Total soluble protein-N** | | **Total soluble amino acid-N** | | **Total soluble protein-N** | |
|  | ***Pseudo-F*** | ***P (perm)*** | ***Pseudo-F*** | ***P (perm)*** | ***Pseudo-F*** | ***P (perm)*** | ***Pseudo-F*** | ***P (perm)*** |
| ***Fagus*** | | | | | | | | |
| Soil N availability | 10.256 | **0.005** | 1.274 | 0.276 | 5.047 | **0.035** | 3.585 | 0.073 |
| Competitor identity | 0.005 | 0.949 | 0.184 | 0.676 | 1.711 | 0.212 | 1.005 | 0.334 |
| Soil N availability x Competitor identity | 0.031 | 0.871 | 1.656 | 0.208 | 1.569 | 0.234 | 3.317 | 0.083 |
| ***Quercus*** | | | | | | | | |
| Soil N availability | 0.152 | 0.701 | 0.165 | 0.689 | 8.155 | 0.008 | 4.520 | **0.046** |
| Competitor identity | 0.016 | 0.901 | 1.789 | 0.197 | 0.843 | 0.375 | 1.044 | 0.325 |
| Soil N availability x Competitor identity | 1.365 | 0.264 | 0.615 | 0.442 | 4.525 | **0.039** | 0.434 | 0.525 |
| ***Pinus*** | | | | | | | | |
| Soil N availability | 23.924 | **<0.001** | 0.606 | 0.439 | 2.420 | 0.134 | 1.957 | 0.177 |
| Competitor identity | 0.294 | 0.594 | 0.181 | 0.664 | 1.654 | 0.205 | 1.596 | 0.226 |
| Soil N availability x Competitor identity | 0.526 | 0.483 | 0.081 | 0.779 | 1.261 | 0.276 | 0.239 | 0.638 |
| ***Prunus*** | | | | | | | | |
| Soil N availability | 5.951 | **0.022** | 4.614 | **0.044** | 8.319 | **0.006** | 1.698 | 0.203 |
| Competitor identity | 1.309 | 0.284 | 0.173 | 0.844 | 0.578 | 0.566 | 2.658 | 0.080 |
| Soil N availability x Competitor identity | 0.348 | 0.697 | 1.730 | 0.193 | 0.677 | 0.514 | 0.652 | 0.518 |
| ***Robinia*** | | | | | | | | |
| Soil N availability | 8.542 | **0.006** | 0.580 | 0.460 | 31.308 | <0.001 | 20.491 | **<0.001** |
| Competitor identity | 0.586 | 0.602 | 1.720 | 0.120 | 6.202 | 0.005 | 0.850 | 0.445 |
| Soil N availability x Competitor identity | 0.550 | 0.625 | 0.345 | 0.714 | 7.590 | **0.001** | 0.362 | 0.710 |

**Supplementary Table 8B.** Pairwise PERMANOVA comparisons of the effects of high compared to low soil N availability and competitor identity on the total amino acid-N (mg amino acid-N g^-1^ dw) levels in fine roots of *Quercus robur* and *Robinia pseudoacacia* seedlings grown in competition, based on significant results from previous main tests (see Table 2A). Significant values (p ≤ 0.05) are indicated in bold.

|  |  | ***t*** | ***P (perm)*** |
| --- | --- | --- | --- |
| ***Quercus*** | | | |
| Soil N availability |  |  |  |
| Low N | *Quercus* vs. *Prunus* - *Quercus* vs. *Robinia* | 1.411 | 0.208 |
| High N | *Quercus* vs. *Prunus* - *Quercus* vs. *Robinia* | 2.580 | **0.007** |
| Competitor identity |  |  |  |
| *Q. robur* vs. *P. serotina* | Low N - High N | 3.120 | **0.004** |
| *Q. robur* vs. *R. pseudoacacia* | Low N - High N | 0.723 | 0.509 |
| ***Robinia*** | | | |
| Soil N availability |  |  |  |
| Low N | *Fagus* vs. *Robinia* - *Quercus* vs. *Robinia* | 2.888 | 0.022 |
|  | *Fagus* vs. *Robinia* - *Pinus* vs. *Robinia* | 3.003 | **0.025** |
|  | *Quercus* vs. *Robinia* - *Pinus* vs. *Robinia* | 0.337 | 0.731 |
| High N | *Fagus* vs. *Robinia* - *Quercus* vs. *Robinia* | 0.181 | 0.861 |
|  | *Fagus* vs. *Robinia* - *Pinus* vs. *Robinia* | 1.087 | 0.340 |
|  | *Quercus* vs. *Robinia* - *Pinus* vs. *Robinia* | 0.935 | 0.447 |
| Competitor identity |  |  |  |
| *Fagus* vs. *Robinia* | Low N - High N | 4.654 | **0.002** |
| *Quercus* vs. *Robinia* | Low N - High N | 3.016 | **0.010** |
| *Pinus* vs. *Robinia* | Low N - High N | 1.591 | 0.109 |
